# Control of cell state transitions by post-transcriptional regulation

**DOI:** 10.1098/rstb.2023.0050

**Published:** 2024-04-22

**Authors:** Carla Mulas

**Affiliations:** Altos Labs Cambridge Institute of Science, Granta Park, Cambridge, CB21 6GP, UK

**Keywords:** cell states, transitions, differentiation

## Abstract

Cell state transitions are prevalent in biology, playing a fundamental role in development, homeostasis and repair. Dysregulation of cell state transitions can lead to or occur in a wide range of diseases. In this letter, I explore and highlight the role of post-transcriptional regulatory mechanisms in determining the dynamics of cell state transitions. I propose that regulation of protein levels after transcription provides an under-appreciated regulatory route to obtain fast and sharp transitions between distinct cell states.

This article is part of a discussion meeting issue ‘Causes and consequences of stochastic processes in development and disease’.

## 1. Introduction

The ability for cells to transition between distinct cellular states is a fundamental feature of biological systems. It underpins the ability of cells to specialize and differentiate during embryonic development and in tissue homeostasis. It is also necessary to respond and adapt to changes in the environment that might result from injury, infections, or other perturbations. Finally, dysregulation is associated with diseases such as developmental disorders or cancers, and cellular states are therefore tightly controlled at multiple levels.

To begin to understand cell state transitions, it is necessary to establish a theoretical framework and clear definitions. In recent years, cell states have mostly been defined molecularly from the ‘bottom-up’ by cataloguing various cellular components [1]. Cells are parametrized by the expression levels of genes, a specific chromatin architecture or metabolic composition. As such, cell states result from the somewhat arbitrary clustering of cells based on molecular similarities [1]. This purely molecular definition of cell states can be used to map the trajectory across a transition [2]. However, such definitions offer limited mechanistic insight as it is difficult to determine when cells have actually transitioned between distinct states or which of these parameters might be functionally important [3].

This has led us to the view that a functional cell state cannot be explained only by cataloguing its components. Instead, we support the idea that a cell state is an emergent property resulting from the combined activity of molecules acting across regulatory levels. Functional studies can be used to measure changes in these emergent properties, such as differentiation capacity, migration or response to electrical stimuli [4]. In this framework, therefore, a cell state is defined by the specific output to a functional test and cell state transition is the process by which the response to that given functional test changes over time. An example of such a functional translation is the switch between the naive and formative state of pluripotency in mouse embryonic stem cells [5]. The key functional tests for naive pluripotency are the ability to survive and form colonies in defined culture conditions (termed 2iLIF) and the capacity to efficiently colonize the epiblast of pre-implantation embryos [6]. As cells transition from a naive to a formative cell state, cells lose both abilities [6,7]. By contrast, the formative cell state is characterized by the ability to respond directly to lineage inductive cues to differentiate, something that naive cells cannot do directly [7–9]. Transitioning between distinct cellular states involves rewiring of the regulatory network controlling the cell state. The rewiring is often associated with changes across regulatory scales, from chromatin to transcription and translation, to metabolism. Within the myriad of molecules that change expression, localization or activity, multiple systems show evidence of a hierarchy: certain determinants are necessary and/or sufficient to instil and maintain a given functional behaviour and therefore identity, whereas other factors alter as a secondary consequence and are redundant. The ability of such determinants to re-instate or drive towards a new identity when overexpressed during somatic cell reprogramming is a fundamental demonstration of this hierarchy [10,11]. Since these determinants can have such a powerful role in maintaining or inducing a cell state, understanding their regulation and the dynamics of their expression and/or activity is fundamental to understanding how cells transition between states. Equally, dysregulation and/or altered dynamics may contribute to pathogenesis. In this letter, I summarize observations on the predicted dynamics of cell state regulators and discuss the implications for cell state transitions.

## 2. Effect of synthesis and degradation rates on transitions

Examining published literature shows that the synthesis rates for both mRNA and protein are much faster than the respective degradation rates. The difference in timescales between synthesis and degradation is a global phenomenon, even when we account for a delay between transcription and translation due to mRNA processing and transport [12]. In mammalian systems, for example, RNA polymerase II has a mean elongation rate of 0.5–3.5 kb min^−1^, depending on the extent of pausing [13–15], meaning that most genes can be synthesized in minutes. Conversely, the median half-life of mRNAs is 3–11 h [16,17].

This difference in synthesis and degradation rates for proteins is even more striking. The ribosome has an average rate of amino acid incorporation of approximately 300 amino acids min^−1^ [18,19]. Therefore, on average, it would take minutes to synthesize a new protein. Conversely, degradation rates are much slower and variable with the half-life of proteins typically ranging from hours to days [16,20]. Although transcriptional regulators are generally less stable and their expression more dynamic [16,21,22], the difference in timescale between synthesis and degradation remains significant.

**Figure 1.**
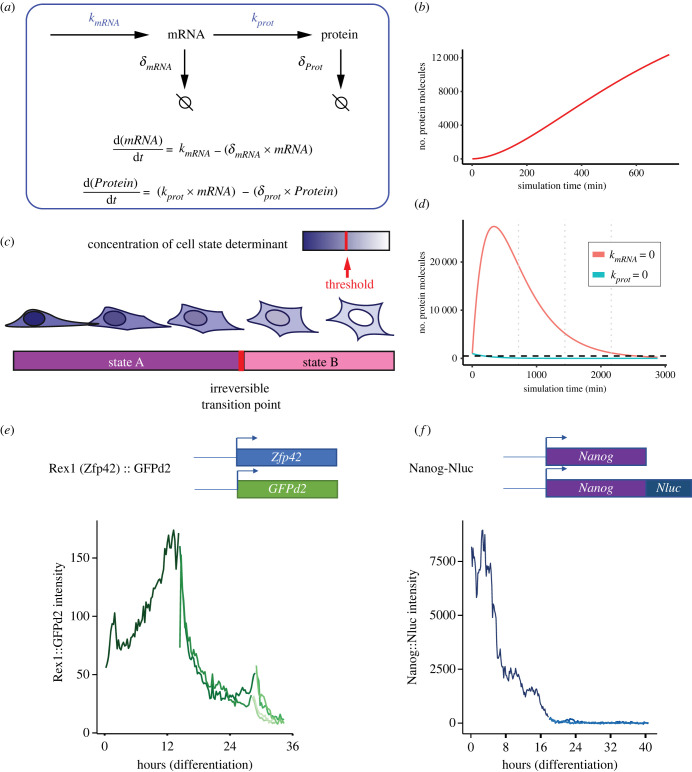
(*a*) Mathematical model showing the key rate parameters controlling the expression of a cell state determinant. The following parameters were used for the simulations: initial Nanog protein levels = 1000 molecules (general number of transcription factor molecules per cell from Biggin [23]), initial *Nanog* mRNA levels = 240 molecules [24]*, Nanog k*_mRNA_ = 0.19 molecules min^−1^ (average transcription rate calculated from: mean number of mRNA produced during an ‘ON’ period (=123 molecules) / mean ‘ON’ duration (144 min) * fraction of time ‘ON’ (0.22)—from Skinner *et al.* [25]), Nanog *δ*_mRNA_ = 0.0022 molecules min^−1^ [25], *k*_prot_ = 0.91 molecules min^−1^ (estimated from the rate of protein translation in mouse embryonic stem cells from Ingolia *et al.* [18]), Nanog *δ*_prot_ = 0.0038 molecules min^−1^ (experimentally determined, [26]). (*b*) Simulation of the number of protein molecules of a cell state determinant over time when all rates are constant. (*c*) Schematic of a differentiating cell, showing decreasing expression of a cell state determinant and the threshold, below which the cell undergoes an irreversible cell state transition. (*d*) Modelling potentially extreme scenarios for the downregulation dynamics of a cell state determinant. Dotted lines show the threshold at which cells undergo irreversible transitions. Vertical lines mark 720 min intervals (average length of cell cycle in mESCs). (*e,f*) Schematic of transcriptional (Rex1::GFPd2, RGd2) and protein fusion (Nanog-Nluc) reporters, showing representative traces of a single cell as it differentiates over time. Different colour shades indicate sister cells after division.

To better demonstrate the implications of the differences in timescales, I here employ a very simplified mathematical model. The model allows us to investigate changes in protein level over time as a function of synthesis and degradation rates of the mRNA and protein. To ground the model within biologically plausible parameters, I have populated the model using rate constants for the transcription factor Nanog (figure 1*a*). Nanog clearance is the rate limiting factor during exit from the embryonic stem cell state in mouse [26] and overexpression significantly delays/impairs the transition to a differentiated state [27,28]. Therefore, it is likely that the rate parameters of Nanog are optimized for transitions and are a good starting point.

From the simulations it can be observed that protein levels increase over time under steady state conditions (figure 1*b*). This predicted increase is consistent with live imaging of Nanog fusion reporters and other proteins over the course of a cell cycle [20], and is moreover consistent with Nanog being synthesized more quickly than it can be cleared when we do not consider division.

What is the implication of the difference in timescales for cell state transitions and the clearance of cell state determinants? Let us assume that a cell state transition occurs when a cell state determinant is downregulated and its concentration goes below a certain threshold (figure 1*c*). We can examine how changes in the rate parameters leads to clearance of the determinant. Specifically, we can measure how long it takes for the determinant to fall below a threshold, since this determines the rate of transitions (figure 1*c*).

To model the most extreme case scenarios, we can look at the changes in protein levels when either the rate of transcription or translation are set to zero. Examining the downregulation kinetics of the determinant undergoing a hypothetical transcriptional switch (transcription rate is zero) shows that this extreme scenario has the slowest transition time, since it would take the longest time for the concentration of the determinant to fall below the threshold (figure 1*d*, red line). A gradual decrease in the rate of mRNA synthesis would lead to an even longer and shallower clearance of a cell state determinant and very long cell state transition times. The overall downregulation kinetics would be influenced by the rate of both mRNA and protein degradation. Two noticeable features characterize transcriptional switches. First, we would predict that shortly after the change in rates (start of a potential transition), protein levels would temporarily continue to increase, as mRNA already present continues to be translated until mRNA degradation catches on. Second, the overall rate of decrease of the determinant should be slower than the actual half-life of the protein.

If instead we model a translation switch by turning the rate of translation to zero, we obtain the fastest clearance (by 13x) of cell state determinants (time taken to cross the threshold) (figure 1*d*, blue line). In such systems, the *rate limiting* factor for cell state transitions becomes exclusively the rate of protein degradation.

We can examine previous data to determine whether the dynamics of different reporters *qualitatively* recapitulate the behaviours of this simple model. For example, the RGd2 reporter is widely used to mark undifferentiated mouse embryonic stem cells (mESCs), and its downregulation marks commitment to differentiation [6]. RGd2 is a classical transcriptional reporter, in which the coding sequence of one allele of *Zfp42* (encoding for the protein Rex1) is replaced with a destabilized form of GFP [29]. Blocking protein synthesis with cycloheximide and measuring the decay in GFP intensity in RGd2 reporter cells over time, shows that the GFPd2 fluorescent protein has a half-life of approximately 2.5 h (figure 1*e*). Since blocking protein synthesis can also inhibit protein degradation by interfering with autophagy [30], this is likely to be a slightly high estimate. However, when we quantified the downregulation dynamics of GFP during ESC differentiation using live imaging, single cells reach 50% of their fluorescent levels around approximately 9 h after initiating downregulation. Therefore, downregulation of the reporter occurs much slower than what we would expect if protein synthesis was inhibited. This supports the idea that downregulation of the GFP reporter occurs at the transcriptional level by changes in mRNA synthesis. This is not surprising since there is no reason to expect post-transcriptional regulation of the GFP protein.

We also examined the dynamics of Nanog protein downregulation using a protein fusion reporter [26]. In this case, the sequence of a bioluminescent reporter is inserted in-frame with the *Nanog* coding sequence before the stop codon, meaning it should reflect both the transcriptional and post-transcriptional regulation on Nanog (figure 1*f*). We inhibited protein synthesis and determined the half-life of the fusion reporter to be 3.02 h in mouse embryonic stem cells which is in general agreement with other protein-fusion reporter systems [31]. Next, we used bioluminescence live imaging [32] to measure the dynamics of Nanog downregulation during differentiation [26]. Surprisingly, we saw that once Nanog started to be downregulated, it reached 50% of its original fluorescent levels in approximately 3.2 h. This means that Nanog downregulation is occurring much closer to the rate limit (given by the protein half-life), compared to what is observed for a purely transcriptional reporter like RGd2. This suggests that the downregulation of Nanog is likely to be controlled at least in part at the post-transcriptional level. Even if transcription was set to zero, protein degradation would have to increase by more than 65 fold (protein half-life of approx. 3 min) for Nanog to be downregulated at the observed rate. Therefore, given the difference in the magnitude of the rate of protein synthesis and degradation (minutes to hours), it is likely that downregulation of Nanog requires either a drastic change in translation or changes in the mRNA available for translation.

In agreement with these observations, it has been noted that regulatory proteins that are under transcriptional control have extremely short half-lives. For example, the Hes1 transcription factor, which is essential for somite segmentation and therefore correct organization of vertebrae and ribs [33], has a half-life of only 20 min [34]. Increasing the Hes1 protein half-life from 20 min to 30 min is enough to disrupt the somite segmentation clock and result in severe skeletal malformations [35].

In our experience the importance of post-transcriptional regulatory mechanisms is perhaps under-appreciated, particularly in an age when single-cell transcriptomics is routinely used to designate cell states. In part, this is due to the technical limitations of imaging protein and RNA dynamics *in vivo* over the course of cell state transitions. It might be possible to detect clues to possible post-transcriptional regulatory mechanisms if they affect mRNA levels. However, most other mechanisms remain difficult to detect by transcriptional readouts alone. Therefore, it is likely that post-transcriptional control of cell state transitions is more prevalent than currently appreciated [36–38].

Multiple regulatory mechanisms have been identified that could potentially lead to rapid changes in protein levels or activity during cell state transitions.

(i) Changes in mRNA stability and availability. MicroRNAs can lead to reduced protein levels without changing translation efficiency [39]. MicroRNA have been shown to be required for the establishment of sharp and robust mutually exclusive pattern of Hoxa5 and Hoxc8 protein expression during motor neurone specification in the spinal cord [40]. Similarly, RNA modifications such as m6A tend to decrease the mRNA half-life [41]. Expression of alternative transcript isoforms with alternative 3′ UTRs can also lead to effective changes in mRNA stability [42]. Changes in splicing can also lead to the removal of poison introns or exons that target transcripts for nonsense mediated decay [43]. Alternatively, mRNAs might be sequestered to prevent their translation. In *Drosophila* intestinal stem cells, P-bodies sequester pro-differentiation mRNAs and prevent their precocious translation and differentiation [44]. It has also been well established that neurones tightly control mRNA localization ensuring that protein translation occurs at very specific sites [45,46] and in response to specific stimuli [45,47].
(ii) Changes in mRNA translation efficiency. Both mRNA elements and environmental conditions have been known to regulate the efficiency of translation. For example, the presence/absence of upstream open reading frames (uORFs) in mRNA isoforms can significantly alter translation efficiency [48]. Similarly, the presence of TOP elements in the 5′UTR of mRNAs can make the translation of mRNAs mTOR dependent [49].
(iii) Changes in protein modification and localization. If the activity of a cell state determinant relies on its nuclear localization, cytoplasmic sequestration is as efficient a mechanism as protein degradation [50]. Equally, post-translational modifications that result in changes of conformation and loss of vital protein–protein interactions can act as effective switches.
(iv) Selective and rapid protein degradation as illustrated during the transition across cell cycle phases [51].

The observation that transcription factors, which often act as cell state determinants, are among the most unstable proteins [16,21,22], supports the idea that protein degradation is the rate limiting factor for cell state transitions. This ultimate dependence of transition time on the rate of protein degradation is also supported by the observation that global differences in protein degradation are responsible for differences in developmental timing across species [52,53]. However, for a biological transition to operate close to the limit rate, I hypothesize that more mechanisms must be in place to stop or significantly reduce the production of new proteins. This remains an understudied area of research. Given the advances in imaging, genetic engineering, and *in vitro* culture systems, more and more experimental systems are becoming accessible. Overall, I think it is likely that post-transcriptional control of cell state transitions is a highly prevalent mechanism and an exciting avenue for further study.

## Data Availability

This article has no additional data.

## References

[1] Stuart T, Satija R. 2019 Integrative single-cell analysis. Nat. Rev. Genet. **20**, 257-272. (10.1038/s41576-019-0093-7) 30696980

[2] Saelens W, Cannoodt R, Todorov H, Saeys Y. 2019 A comparison of single-cell trajectory inference methods. Nat. Biotechnol. **37**, 547-554. (10.1038/s41587-019-0071-9) 30936559

[3] Weinreb C, Rodriguez-Fraticelli A, Camargo FD, Klein AM. 2020 Lineage tracing on transcriptional landscapes links state to fate during differentiation. Science **367**, eaaw3381. (10.1126/science.aaw3381) 31974159 PMC7608074

[4] Mulas C, Chaigne A, Smith A, Chalut KJ. 2021 Cell state transitions: definitions and challenges. Development **148**, dev199950. (10.1242/dev.199950) 34932803

[5] Smith A. 2017 Formative pluripotency: the executive phase in a developmental continuum. Development **144**, 365-373. (10.1242/dev.142679) 28143843 PMC5430734

[6] Kalkan T et al. 2017 Tracking the embryonic stem cell transition from ground state pluripotency. Development **144**, 1221-1234. (10.1242/dev.142711) 28174249 PMC5399622

[7] Kinoshita M, Barber M, Mansfield W, Cui Y, Spindlow D, Stirparo GG, Dietmann S, Nichols J, Smith A. 2021 Capture of mouse and human stem cells with features of formative pluripotency. Cell Stem Cell **28**, 453-471.e8. (10.1016/j.stem.2020.11.005) 33271069 PMC7939546

[8] Hayashi K, Ohta H, Kurimoto K, Aramaki S, Saitou M. 2011 Reconstitution of the mouse germ cell specification pathway in culture by pluripotent stem cells. Cell **146**, 519-532. (10.1016/j.cell.2011.06.052) 21820164

[9] Mulas C, Kalkan T, Smith A. 2017 NODAL secures pluripotency upon embryonic stem cell progression from the ground state. Stem Cell Reports **9**, 77-91. (10.1016/j.stemcr.2017.05.033) 28669603 PMC5511111

[10] Takahashi K, Yamanaka S. 2016 A decade of transcription factor-mediated reprogramming to pluripotency. Nat. Rev. Mol. Cell Biol. **17**, 183-193. (10.1038/nrm.2016.8) 26883003

[11] Wang H, Yang Y, Liu J, Qian L. 2021 Direct cell reprogramming: approaches, mechanisms and progress. Nat. Rev. Mol. Cell Biol. **22**, 410-424. (10.1038/s41580-021-00335-z) 33619373 PMC8161510

[12] Schott J et al. 2021 Nascent Ribo-Seq measures ribosomal loading time and reveals kinetic impact on ribosome density. Nat. Methods **18**, 1068-1074. (10.1038/s41592-021-01250-z) 34480152

[13] Darzacq X, Shav-Tal Y, Turris Vd, Brody Y, Shenoy SM, Phair RD, Singer RH. 2007 *In vivo* dynamics of RNA polymerase II transcription. Nat. Struct. Mol. Biol. **14**, 796-806. (10.1038/nsmb1280) 17676063 PMC4942130

[14] Jonkers I, Kwak H, Lis JT. 2014 Genome-wide dynamics of Pol II elongation and its interplay with promoter proximal pausing, chromatin, and exons. eLife **3**, e02407. (10.7554/eLife.02407) 24843027 PMC4001325

[15] Swinburne IA, Silver PA. 2008 Intron delays and transcriptional timing during development. Dev. Cell **14**, 324-330. (10.1016/j.devcel.2008.02.002) 18331713 PMC2825037

[16] Schwanhäusser B, Busse D, Li N, Dittmar G, Schuchhardt J, Wolf J, Chen W, Selbach M. 2011 Global quantification of mammalian gene expression control. Nature **473**, 337-342. (10.1038/nature10098) 21593866

[17] Sharova LV, Sharov AA, Nedorezov T, Piao Y, Shaik N, Ko MSH. 2009 Database for mRNA half-life of 19 977 genes obtained by DNA microarray analysis of pluripotent and differentiating mouse embryonic stem cells. DNA Res. **16**, 45-58. (10.1093/dnares/dsn030) 19001483 PMC2644350

[18] Ingolia NT, Lareau LF, Weissman JS. 2011 Ribosome profiling of mouse embryonic stem cells reveals the complexity and dynamics of mammalian proteomes. Cell **147**, 789-802. (10.1016/j.cell.2011.10.002) 22056041 PMC3225288

[19] Yan X, Hoek TA, Vale RD, Tanenbaum ME. 2016 Dynamics of translation of single mRNA molecules *in vivo*. Cell **165**, 976-989. (10.1016/j.cell.2016.04.034) 27153498 PMC4889334

[20] Alber AB, Suter DM. 2019 Dynamics of protein synthesis and degradation through the cell cycle. Cell Cycle **18**, 784-794. (10.1080/15384101.2019.1598725) 30907235 PMC6527273

[21] Alber AB, Paquet ER, Biserni M, Naef F, Suter DM. 2018 Single live cell monitoring of protein turnover reveals intercellular variability and cell-cycle dependence of degradation rates. Mol. Cell **71**, 1079-1091.e9. (10.1016/j.molcel.2018.07.023) 30146318

[22] Li J, Cai Z, Vaites LP, Shen N, Mitchell DC, Huttlin EL, Paulo JA, Harry BL, Gygi SP. 2021 Proteome-wide mapping of short-lived proteins in human cells. Mol. Cell **81**, 4722-4735.e5. (10.1016/j.molcel.2021.09.015) 34626566 PMC8892350

[23] Biggin MD. 2011 Animal transcription networks as highly connected, quantitative continua. Dev. Cell **21**, 611-626. (10.1016/j.devcel.2011.09.008) 22014521

[24] Ochiai H, Sugawara T, Sakuma T, Yamamoto T. 2014 Stochastic promoter activation affects Nanog expression variability in mouse embryonic stem cells. Sci. Rep. **4**, 7125. (10.1038/srep07125) 25410303 PMC4238020

[25] Skinner SO, Xu H, Nagarkar-Jaiswal S, Freire PR, Zwaka TP, Golding I. 2016 Single-cell analysis of transcription kinetics across the cell cycle. eLife **5**, e12175. (10.7554/eLife.12175) 26824388 PMC4801054

[26] Mulas C, Stammers M, Salomaa SI, Heinzen C, Suter DM, Smith A, Chalut KJ. 2023 ERK signalling orchestrates metachronous transition from naïve to formative pluripotency. *bioRxiv* 2023.07.20.549835. (10.1101/2023.07.20.549835) 39069943

[27] Carbognin E et al. 2023 Esrrb guides naive pluripotent cells through the formative transcriptional programme. Nat. Cell Biol. **25**, 643-657. (10.1038/s41556-023-01131-x) 37106060 PMC7614557

[28] Chambers I, Colby D, Robertson M, Nichols J, Lee S, Tweedie S, Smith A. 2003 Functional expression cloning of Nanog, a pluripotency sustaining factor in embryonic stem cells. Cell **113**, 643-655. (10.1016/S0092-8674(03)00392-1) 12787505

[29] Li X, Zhao X, Fang Y, Jiang X, Duong T, Fan C, Huang C-C, Kain SR. 1998 Generation of destabilized green fluorescent protein as a transcription reporter. J. Biol. Chem. **273**, 34 970-34 975. (10.1074/jbc.273.52.34970) 9857028

[30] Watanabe-Asano T, Kuma A, Mizushima N. 2014 Cycloheximide inhibits starvation-induced autophagy through mTORC1 activation. Biochem. Biophys. Res. Commun. **445**, 334-339. (10.1016/j.bbrc.2014.01.180) 24525133

[31] Filipczyk A et al. 2015 Network plasticity of pluripotency transcription factors in embryonic stem cells. Nat. Cell Biol. **17**, 1235-1246. (10.1038/ncb3237) 26389663

[32] Mandic A, Strebinger D, Regali C, Phillips NE, Suter DM. 2017 A novel method for quantitative measurements of gene expression in single living cells. Methods **120**, 65-75. (10.1016/j.ymeth.2017.04.008) 28456689

[33] Bessho Y, Sakata R, Komatsu S, Shiota K, Yamada S, Kageyama R. 2001 Dynamic expression and essential functions of Hes7 in somite segmentation. Genes Dev. **15**, 2642-2647. (10.1101/gad.930601) 11641270 PMC312810

[34] Bessho Y, Hirata H, Masamizu Y, Kageyama R. 2003 Periodic repression by the bHLH factor Hes7 is an essential mechanism for the somite segmentation clock. Genes Dev. **17**, 1451-1456. (10.1101/gad.1092303) 12783854 PMC196074

[35] Hirata H, Bessho Y, Kokubu H, Masamizu Y, Yamada S, Lewis J, Kageyama R. 2004 Instability of Hes7 protein is crucial for the somite segmentation clock. Nat. Genet. **36**, 750-754. (10.1038/ng1372) 15170214

[36] Saba JA, Liakath-Ali K, Green R, Watt FM. 2021 Translational control of stem cell function. Nat. Rev. Mol. Cell Biol. **22**, 671-690. (10.1038/s41580-021-00386-2) 34272502

[37] Tahmasebi S, Amiri M, Sonenberg N. 2019 Translational control in stem cells. Front. Genetics **9**, 709. (10.3389/fgene.2018.00709) PMC6341023 30697227

[38] Wang R, Amoyel M. 2022 mRNA translation is dynamically regulated to instruct stem cell fate. Front. Mol. Biosci. **9**, 863885. (10.3389/fmolb.2022.863885) 35433828 PMC9008482

[39] Guo H, Ingolia NT, Weissman JS, Bartel DP. 2010 Mammalian microRNAs predominantly act to decrease target mRNA levels. Nature **466**, 835-840. (10.1038/nature09267) 20703300 PMC2990499

[40] Li C et al. 2021 MicroRNA governs bistable cell differentiation and lineage segregation via a noncanonical feedback. Mol. Syst. Biol. **17**, e9945. (10.15252/msb.20209945) 33890404 PMC8062999

[41] Wang X et al. 2014 N6-methyladenosine-dependent regulation of messenger RNA stability. Nature **505**, 117-120. (10.1038/nature12730) 24284625 PMC3877715

[42] Tushev G, Glock C, Heumüller M, Biever A, Jovanovic M, Schuman EM. 2018 Alternative 3′ UTRs modify the localization, regulatory potential, stability, and plasticity of mRNAs in neuronal compartments. Neuron **98**, 495-511.e6. (10.1016/j.neuron.2018.03.030) 29656876

[43] Leclair NK et al. 2020 Poison exon splicing regulates a coordinated network of SR protein expression during differentiation and tumorigenesis. Mol. Cell **80**, 648-665.e9. (10.1016/j.molcel.2020.10.019) 33176162 PMC7680420

[44] Buddika K et al. 2022 Coordinated repression of pro-differentiation genes via P-bodies and transcription maintains *Drosophila* intestinal stem cell identity. Curr. Biol. **32**, 386-397.e6. (10.1016/j.cub.2021.11.032) 34875230 PMC8792327

[45] Biever A, Donlin-Asp PG, Schuman EM. 2019 Local translation in neuronal processes. Curr. Opin. Neurobiol. **57**, 141-148. (10.1016/j.conb.2019.02.008) 30861464

[46] Zappulo A et al. 2017 RNA localization is a key determinant of neurite-enriched proteome. Nat. Commun. **8**, 583. (10.1038/s41467-017-00690-6) 28928394 PMC5605627

[47] Younts TJ, Monday HR, Dudok B, Klein ME, Jordan BA, Katona I, Castillo PE. 2016 Presynaptic protein synthesis is required for long-term plasticity of GABA release. Neuron **92**, 479-492. (10.1016/j.neuron.2016.09.040) 27764673 PMC5119541

[48] Cheng Z, Otto GM, Powers EN, Keskin A, Mertins P, Carr SA, Jovanovic M, Brar GA. 2018 Pervasive, coordinated protein-level changes driven by transcript isoform switching during meiosis. Cell **172**, 910-923.e16. (10.1016/j.cell.2018.01.035) 29474919 PMC5826577

[49] Umegaki Y, Brotons AM, Nakanishi Y, Luo Z, Zhang H, Bonni A, Ikeuchi Y. 2018 Palladin is a neuron-specific translational target of mTOR signaling that regulates axon morphogenesis. J. Neurosci. **38**, 4985-4995. (10.1523/JNEUROSCI.2370-17.2018) 29712777 PMC5966794

[50] Betschinger J, Nichols J, Dietmann S, Corrin PD, Paddison PJ, Smith A. 2013 Exit from pluripotency is gated by intracellular redistribution of the bHLH transcription factor Tfe3. Cell **153**, 335-347. (10.1016/j.cell.2013.03.012) 23582324 PMC3661979

[51] Dang F, Nie L, Wei W. 2021 Ubiquitin signaling in cell cycle control and tumorigenesis. Cell Death Differ. **28**, 427-438. (10.1038/s41418-020-00648-0) 33130827 PMC7862229

[52] Matsuda M et al. 2020 Species-specific segmentation clock periods are due to differential biochemical reaction speeds. Science **369**, 1450-1455. (10.1126/science.aba7668) 32943519

[53] Rayon T et al. 2020 Species-specific pace of development is associated with differences in protein stability. Science **369**, eaba7667. (10.1126/science.aba7667) 32943498 PMC7116327

